# A Machine Learning Approach as a Surrogate for a Finite Element Analysis: Status of Research and Application to One Dimensional Systems

**DOI:** 10.3390/s21051654

**Published:** 2021-02-27

**Authors:** Poojitha Vurtur Badarinath, Maria Chierichetti, Fatemeh Davoudi Kakhki

**Affiliations:** 1 Computer Engineering Department, San Jose’ State University, San Jose, CA 95192, USA; poojitha.vurturbadarinath@sjsu.edu; 2 Department of Aerospace Engineering, San Jose’ State University, San Jose, CA 95192, USA; 3 Machine Learning and Safety Analytics Lab, Department of Technology, San Jose’ State University, San Jose, CA 95192, USA; fatemeh.davoudi@sjsu.edu

**Keywords:** finite element, beam analysis, structural monitoring, machine learning, random forest trees, artificial neural networks, gradient boosting regression trees

## Abstract

Current maintenance intervals of mechanical systems are scheduled a priori based on the life of the system, resulting in expensive maintenance scheduling, and often undermining the safety of passengers. Going forward, the actual usage of a vehicle will be used to predict stresses in its structure, and therefore, to define a specific maintenance scheduling. Machine learning (ML) algorithms can be used to map a reduced set of data coming from real-time measurements of a structure into a detailed/high-fidelity finite element analysis (FEA) model of the same system. As a result, the FEA-based ML approach will directly estimate the stress distribution over the entire system during operations, thus improving the ability to define ad-hoc, safe, and efficient maintenance procedures. The paper initially presents a review of the current state-of-the-art of ML methods applied to finite elements. A surrogate finite element approach based on ML algorithms is also proposed to estimate the time-varying response of a one-dimensional beam. Several ML regression models, such as decision trees and artificial neural networks, have been developed, and their performance is compared for direct estimation of the stress distribution over a beam structure. The surrogate finite element models based on ML algorithms are able to estimate the response of the beam accurately, with artificial neural networks providing more accurate results.

## 1. Introduction

Structural health monitoring (SHM) aims at accurately identifying the current state and the behavior of a structure by analyzing data collected through various monitoring devices and sensors over the structure [1,2,3]. Stress analysis is a pivotal part of any mechanical system design. A finite element analysis (FEA) is generally used to perform stress analysis of complex structures and systems for design, maintenance, and safety evaluation across many industries, such as aerospace, automotive, architecture, and biomedical engineering [4,5,6,7]. 

A review of literature, presented in Section 2 of this work, describes preliminary attempts to use machine learning (ML) algorithms in conjunction with finite element analyses, mostly to approach static biomechanical systems. However, the evaluation of the performance of regression ML algorithms in real-time estimation and prediction of stresses in time-varying mechanical systems is a rarely addressed area of research. This study aims to apply, validate and compare the performance of ML methods in accurate estimation of time-varying stresses as a surrogate for an FEA in a one-dimensional framework (beam structure). 

This study contributes to the rare literature on practical applications of ML in stress prediction in an autonomous system by evaluating the performance of artificial neural networks (ANN), random forest decision trees, and optimized gradient boosting trees. The main contribution of this study is proposing the use of supervised ML techniques to build a surrogate finite element model of a time-varying, one-dimensional mechanical system. In addition, the analysis of results provides insights into the challenges and opportunities of creating an ML pipeline as surrogate for an FEA that has broad applications for stress prediction in other industries. The second section of the paper includes a literature review of ML methods and their application for stress prediction purposes. Section 3 includes a discussion of the data set on which the modeling was based on, followed by a brief discussion of the methodology. Section 4 presents, assesses, and compares the results from each regression ML model, that are discussed in Section 5. Conclusions about the performance of the prediction methods, and the application of the proposed analyses in improving a real-time FEA complete the paper in Section 6.

## 2. Machine Learning Modeling for Finite Element Analysis

ML techniques have the ability to automatically generate a model using data from past experiences. The number of applications is extensive and includes self-driving cars, high-frequency trading, house price estimation, search engines, bioinformatics, chemistry, and material science, for which a large amount of data is available [8]. Cases involving large amounts of variables, high levels of uncertainty, and rapid changes in behavior are among the typical scenarios. In this paper, we propose to use supervised ML to generate surrogate models of dynamic systems for accurate stress predictions.

Supervised ML estimates the relationship between some input variables and one or more numeric outputs using optimization algorithms that minimize the approximation error. The error function defines the distance between an output value in the dataset and the output produced by the approximated model. Each particular ML method contains its own error function and optimization algorithm. The optimization process over the data is known as model training. ML algorithms are very powerful predictive tools, but due to their complexity, they generally don’t provide analytical relationships between the input and output data, and are often referred to as “black boxes” as the user loses insight into the underlying physics. The use of ML approaches in conjunction with finite element approaches has recently seen a lot of interest from the research community. The common interest is to improve the trade-off between computational accuracy and computational costs. Before describing the approach proposed in this paper, the authors have identified the main research contributions to the topic, as well as challenges and opportunities in the different research areas.

A few recent attempts exist to use supervised ML algorithms as finite element surrogates to tackle biomechanical problems, such as the stress analysis of the aorta [5,9], the biomechanical behavior of human soft tissue [10], breast tissue [11], and of the human liver [12]. Liang et al. [5] developed patient-specific models of the stress distribution of the aorta using ML algorithms trained using as input the finite element model data and directly outputting the aortic wall stress distribution. The trained model is capable of predicting the stress distributions with errors less than 1% (Von Mises stresses) and demonstrates the feasibility and great potential of using the ML technique as a fast and accurate surrogate finite element for real-time static stress analysis of patient-specific geometries. The authors use an encoding-decoding deep neural network algorithm to model the complex, non-linear relationships between input (anatomical models and finite element results) and output variables (aortic wall stress distribution). Madani et al. [9] develop a finite element surrogate model to compute the aortic wall stress distribution in patients affected by atherosclerosis that learn the underlying mapping between the aortic input parameters (tissue geometry, composition, and arterial pressure) and the output stress distribution, in a highly non-linear environment. Their analyses show that, given a geometry, they can predict real-time the peak von Mises stress magnitude with an average error of less than 10% without the need of highly trained finite element specialists, therefore allowing quick clinical analysis of the patient health. Clinicians can, in fact, use this information during patient examination, evaluation, and treatment for potential areas of high rupture risk. Martinez-Martinez et al. [11] present a data-driven method to simulate, in real-time, the biomechanical behavior of the breast tissues. Decision tree and random forest ML models were trained with data from finite element simulations given the geometry of the tissue. The surrogate model was then used to predict real-time the deformation under compression. A similar approach was used in Reference [12] to predict the biomechanical behavior of the human liver during the breathing process, which is crucial for guiding surgeons during interventions where it is critical to track this deformation. These examples in the biomechanical field show that creating surrogate finite element models using ML is a feasible approach to provide a case-specific approximation of the response of the system (stresses in this case). More validation of the approaches is, however, necessary with a larger sets of data. A major limitation of the approaches [5,9,10,11,12] is their focus on static stress analysis: No time dependency is considered, and only stresses are predicted by the ML approach. In aerospace and mechanical applications, vibrations and time-dependent analysis are of paramount importance, as well as predictions of accelerations, velocities, and displacements in addition to stresses. 

ML has recently been used in computational mechanics to formulate multiscale elements [13,14], enhance the performance of traditional elements [15], and produce data-driven solvers [16]. For example, Capuano et al. [14] use ML techniques to formulate a novel multiscale finite element algorithm, called “Smart Element” that is characterized by low computational cost. The approach utilizes ML to generate a direct relationship between the element state (outputs) and its forces (inputs) and avoids the complex task of finding the internal displacement field, and eliminates the need for numerical iterations. The finite element of interest is fed to an ML algorithm that generates an approximated model (surrogate) of the element that can be used in the same context and that is physically accurate. The approach is general, and it is not tied to any specific ML algorithm because the authors separated the element behavior, the learning process, and the solution method. Therefore, the assembling and solution of the system of equations can be performed using traditional techniques. 

The main challenge in using ML approaches is that the approximation error converges to reasonable values only with a large amount of data, which is often difficult and expensive to obtain for complex numerical models, such as finite elements. To reduce the amount of data needed to train the ML algorithm, Raissi and his team [17,18,19] have developed physics-informed ML approaches. The hypothesis is that if we can encode information based on the underlying physics of the system, less data will be necessary for the ML algorithm to learn. Capuano et al. [14] demonstrate that the use of physics-based information, such as corotational displacements, drastically reduces the samples required for training. Physics-based ML is a promising field, and requires the use of governing partial differential equations to inform the ML algorithm. To the authors’ knowledge, the approach has not been applied using weak-form governing equations, nor more general discretized models, such as finite element models. 

A few attempts also exist in building surrogate modeling of fluid dynamic systems using ML [20,21]. For example, Martinez et al. [21] developed a mid-fidelity approach using an ML surrogate model that can learn the aerodynamic behavior of rotor blades from higher fidelity CFD simulations. The authors obtained an efficient and accurate computational tool that is one order of magnitude faster compared to full CFD simulation and provides superior predictions than traditional lower fidelity approaches. The ML model consists of a Deep Convolutional Neural Network (uses an encoder-decoder architecture with eight convolutional layers and 1024 neuron units) trained with supervised learning techniques on high fidelity CFD simulations which encompass a large range of operating environments for rotor blades. Aero-elastic deformations are initially prescribed as inputs to the ML model that returns the pressure coefficient and skin friction distribution. The paper develops a physics informed neural network (PINN) in which the non-linear partial differential equations that describe the laws of physics for rotorcraft aeromechanics act as a regularization agent that constrains the solution space by discarding any non-realistic flow solutions that violate physical principles. Encoding this information into the learning algorithm enables it to find solutions quicker, as well as improving predictions of unseen data. The predictions are inherently unsteady, three-dimensional, coupled, and highly turbulent, and capture high-dimensional and non-linear observations. Accuracy on aerodynamic loads (normal force and pitching moment) is improved by 97% in the two flight conditions analyzed by the authors (high-speed flight condition and stall flight condition). The analysis is, however, incomplete in the analysis of additional conditions, such as hovering and low-speed flight, and is highly limited by the flight conditions included in the initial dataset. Comparisons on the resulting blade bending and torsional moments are not provided, and therefore, it not possible to conclude the accuracy in structural loads/stresses computation. 

In the domain of geotechnical engineering analysis, Gao et al. [22] use deep learning algorithms (enhance-and-split feature capsule network embedded in fully convolutional neural networks) to generate a surrogate model to predict in-situ static stresses (intrinsic stress of the crust and rock formations in their original state without being disturbed by artificial engineering). The authors use fast Lagrangian analysis based on the finite difference method and the model geometry to train the ML algorithm, and obtain fast and accurate predictions. However, Gao et al. [22] obtain “incomprehensive” features, and remain a promising but not adequate approach yet. 

The review of current work described in this section highlights the lack of investigation of the applicability of surrogate finite elements to transient vibration analysis, which is the main goal of this paper. Based on the time-dependent response at a few reference locations, the ML method will predict the time-dependent response over the spatial domain of the entire beam, as described in the next section. 

## 3. Materials and Methods

This section discusses how training data have been generated, the development of ML models, and the evaluation criteria for the performance of the developed models as a surrogate for an FEA in a one-dimensional time-varying mechanical system. 

### 3.1. Concept

The surrogate FEA approach combines finite element models, supervised ML algorithms, and measured response. Supervised ML is used to create a mid-fidelity surrogate model that learns the stress distribution from rich FEA simulations and predicts stress distributions in real-time based on actual measurements. As the stress of a one-dimensional beam is directly related to the acceleration, the algorithm will focus on predicting overall accelerations. Accelerations are also used as actual real-time measurements because accelerometers are typically and conveniently used to monitor the behavior of a time-varying system. The approach is, however, very flexible, and any type of response variable (displacement, velocity, acceleration, strain, stress) could be used for training and predictions.

The goal of this approach is to obtain a mid-fidelity algorithm that can provide sufficiently accurate numerical predictions of the response of a dynamic system during actual conditions in a very short time [23,24]. The finite element model is informed of the actual condition a time-varying mechanical system is experiencing through the measure response (i.e., acceleration) and the ML algorithm that will evaluate the response of the system at any other location. 

**Figure 1:**
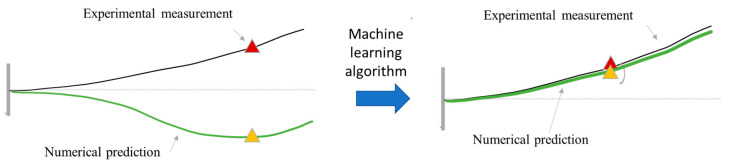
Concept of the approach. On the left, the numerical predictions before the application of the machine learning (ML) approach are not representative of the behavior of the system. After the ML algorithm is applied, the numerical prediction is a good representation of the actual system.

The conceptual vision of this approach is that after training, the surrogate finite element will provide a mid-fidelity estimate of the response of a vehicle at any instant of time during operations, and at any locations of interest (see Figure 1).

To achieve this objective, the following sequence of steps is proposed.

**Figure 2:**
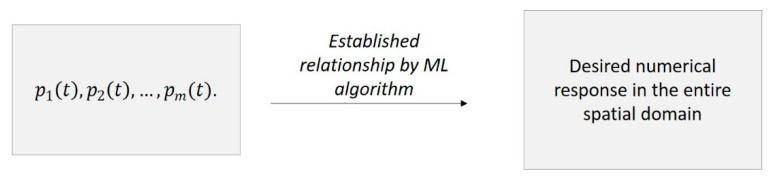
Schematic of ML training approach. Quantities $p_{1}\left(t\right),\;p_{2}\left(t\right),\ldots ,\;p_{m}\left(t\right)$ are evaluated in the finite element model, and the ML model is trained using these quantities as inputs.

1. Construct a finite element model of the system of interest, in the form:
  (1) $$M\ddot{u}\left(t\right)+C\dot{u}\left(t\right)+Ku\left(t\right)=f$$
  where u(t) is a vector containing the nodal degrees of freedom that depends on time t, and $\dot{u}\left(t\right)$ and $\ddot{u}\left(t\right)$ are its first and second derivative in time, respectively. M is the mass matrix, C is the damping matrix, K is the stiffness matrix and f is the vector containing the discretized applied loads for each degree of freedom. The formulation is general and can accommodate for non-linearities in the model.
2. Run a series of time-varying FEA simulations to create a sufficiently large training dataset that represents the expected loading conditions. Several loading conditions ($f_{1},\ldots ,\;f_{n})$ are applied to the model to obtain the response of each degree of freedom and its derivatives $\left(u_{1},\;{\dot{u}}_{1},{\ddot{u}}_{1},\ldots ,\;u_{n},\;{\dot{u}}_{n},{\ddot{u}}_{n}\right)$
  (2) $$\begin{array}{c} f_{1}\to u_{1},\;{\dot{u}}_{1},{\ddot{u}}_{1}\\ \begin{array}{c} f_{2}\to u_{2},\;{\dot{u}}_{2},{\ddot{u}}_{2}\\ \vdots \\ f_{n}\to u_{n},\;{\dot{u}}_{n},{\ddot{u}}_{n} \end{array} \end{array}$$
  The loading conditions can also be applied sequentially to represent continuous variations in the external loads such that
  (3) $$f=[f_{1},\ldots ,\;f_{n}]\to \;u_{},\;{\dot{u}}_{},{\ddot{u}}_{}=[u_{1},\ldots ,u_{n}],\;[{\dot{u}}_{1},\ldots ,\;{\dot{u}}_{n}],\;[{\ddot{u}}_{1},\ldots ,{\ddot{u}}_{n}]$$
  Based on the results, compute stresses S, strain ε and all desired quantities.
3. Identify a few reference locations in the system at which the response will be measured. The quantities at these locations are defined as $p_{1}\left(t\right),\;p_{2}\left(t\right),\ldots ,\;p_{m}\left(t\right)$. Counter m corresponds to the total number of reference locations. Variables $p_{i}\left(t\right)$ can be in the form of strains, accelerations, displacements, velocities, stresses or a combination of them, depending on available sensing capabilities.
4. Set the results of FEA simulations as input to the ML approach. At each time step, the ML algorithm establishes a relationship between the desired numerical response (i.e., strains, accelerations, displacements, velocities, stresses) in the entire domain of the finite element model with the reference quantities $p_{1}\left(t\right),\;p_{2}\left(t\right),\ldots ,\;p_{m}\left(t\right)\;$ computed using the finite element model, Figure 2.
5. Measure the response at the reference locations $p_{1}\left(t\right),\;p_{2}\left(t\right),\ldots ,\;p_{m}\left(t\right)$ and input them to the trained ML model.
6. Evaluate the desired numerical response in the entire spatial domain of the system based on the measured $p_{1}\left(t\right),\;p_{2}\left(t\right),\ldots ,\;p_{m}\left(t\right)$.

### 3.2. Data Generation

The feasibility of a surrogate finite element model is analyzed in a numerical environment, in which both the training and prediction data is created by a finite element solver (corresponding to Steps 3 and 5 of the procedure in Section 3.1). This numerical setting allows for more flexibility in data generation, and less variability, since no experimental errors and noise will be affecting the predictions. The acceleration at five locations along the beam is set as reference variable $p_{1}\left(t\right),\;p_{2}\left(t\right),\ldots ,\;p_{5}\left(t\right)$ and the entire acceleration fields is reconstructed along the span of the system $\ddot{u}$(*t*).

The analysis focuses on one-dimensional, linear, flexible dynamic systems, for which the mechanical properties of the system (material properties and geometry) are known. Specifically, the analysis is based on a rectangular, aluminum beam. The beam is 0.24 m long, 0.032 m wide, and 3 mm thick. It is made of aluminum alloy, with Young modulus E = 70,000 MPa, Poisson ratio ν=0.33 and mass density $\rho =\;2700\;\mathrm{kg}/m^{3}$.

A finite element model of the beam is developed using the material and geometrical properties described in the previous section. A mesh of 40 Timoshenko beam elements is considered, clamped at the base. Light dissipation is introduced in the form of proportional damping, C = αM + βK, with $\alpha =10^{-1}\;1/s$ and $\beta =2\;\times \;10^{-6}\;s$. The time integration is performed using the Newmark scheme (2nd order accuracy), and the finite element code is implemented in Matlab [25,26,27].

**Figure 3:**
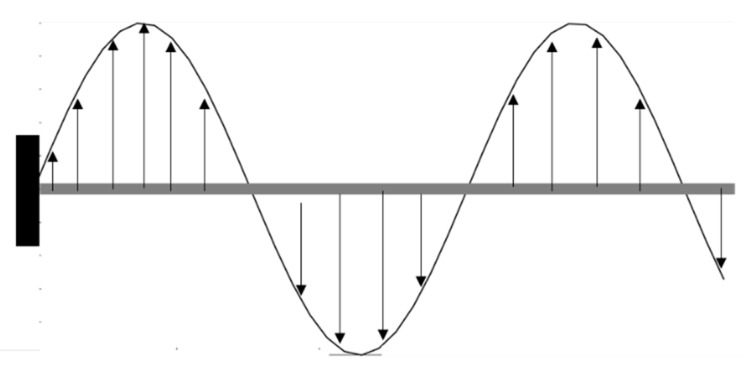
Numerical model of the beam and spatial load distribution for training the ML algorithms.

**Figure 4:**
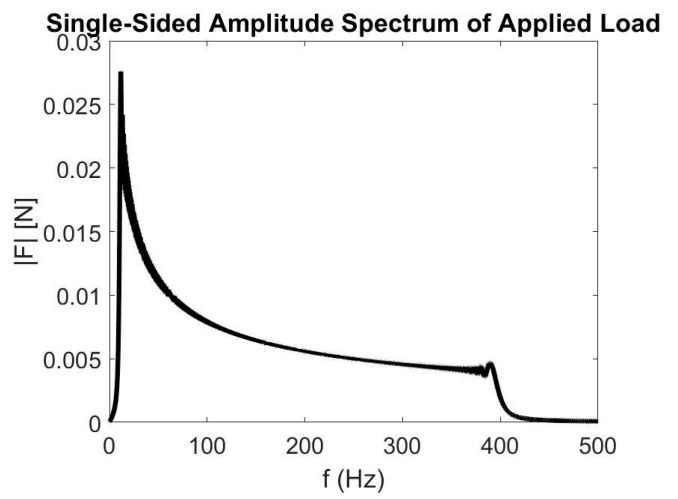
Frequency analysis for load distribution for case 1.

The first bending natural frequency is 28 Hz, and the second 220 Hz. A distributed load is applied along the beam, with a sinusoidal spatial distribution and a sine chirp time variation in frequency range [0–400] Hz. This applied load ensures that the entire frequency range is excited, and that the contribution of a load acting on each node is considered as well. The time response is numerically integrated using $∆t=2.5\;\times 10^{-4}s$ time step to prevent aliasing, and the simulation has a duration of 10 s, for a total of 4 × 10^4^ time steps. The numerical model of the beam and spatial load distribution and the frequency analysis for load distribution are shown in Figure 3 and Figure 4, respectively. Given the variations in the applied loads both in time and space, this loading condition is considered as the entire loading vector f based on which the response of the model is computed.

The results of the finite element model are then organized in a tabular form, and each row contains the information for each nodal location in the beam at each time step. The input variables *p*_1_*, p*_2_*, p*_3_*, p*_4_*, p*_5_ are specified at the following locations along the beam, respectively: *x_pi_* = [0.006, 0.073, 0.121, 0.199, 0.242] m, such as in Table 1.

For a total of 1.64 × 10^6^ rows = (4 × 10^4^ time steps) × (41 nodal locations x). The reference locations x_pi_ have been chosen to have a uniformly spaced distribution of points over the beam domain.

### 3.3. Data Pre-Processing 

The data generated in the previous section is used to develop the ML models. The dataset consists of over 1.6 million data points. The dataset has eight attributes: Time instant at which acceleration is measured (*time*), location at which acceleration is measured (*x*), acceleration (*acc*), and *p*_1_*, p*_2_*, p*_3_*, p*_4_*, p*_5_, each representing acceleration values obtained at different places on the beam when load is applied at variable *x*. All the five accelerations *p*_1_*, p*_2_*, p*_3_*, p*_4_*, p*_5_ are obtained at the same time instance. The purpose is to use acceleration values at a specific *x* location over a setting of time instances to predict acceleration in other points. 

The datatype of all attributes is numerical. None of the attributes has any missing data. Since the experimental data is generated using the FE model, no values are considered as outliers. The time ‘*t*’ attribute is not used for the analysis. The variables used as independent/input attributes are: *x, p*_1_*, p*_2_*, p*_3_*, p*_4_, and *p*_5_; and the output variable to be predicted is *acc*. 

Since the range of attribute values are extremely different, due to the nature of the variation over time for this specific model, the data is normalized. We used the normalization method as the traditional way of scaling data in the ML modeling. That way, the data is secure for modeling as it can now be transformed from its original distribution to the “Normal Z-Distribution”. The formula used by the algorithm is that for each variable ($v_{i})$, its value is deduced from the overall average of the value (μ), and is divided by the overall standard deviation of that variable (σ), using the following equation:

$$Z_{i}=\frac{v_{i}-\mu }{\sigma }$$

Any calculated statistical feature, such as minimum, maximum, standard deviation, and quantiles, naturally inherits the same units of the original variable. Therefore, the unit for the statistical features, presented in Table 2, have all the same units after being normalized. The descriptive statistics of the generated data is shown in Table 2. 

From the whole dataset, 80% of data is used in the training process for developing each ML model separately. Then, the trained model is saved and applied to the new dataset, which includes 20% of the cases in the original data. The purpose is to assure the practicality and efficiency of the model when applied to new cases. As long as the developed ML algorithms could generalize and show a comparable accuracy rate with the training results, the model is useful for prediction purposes. 

### 3.4. Machine Learning Model Development 

In this study, three ML algorithms for regression, including decision trees and artificial neural networks, are used to predict the acceleration based on previously given accelerations in specific time instances and locations over the beam. Decision trees are among the most popular predictive analytics techniques among practitioners, due to being relatively straightforward to build and understand, as well as handling both nominal and continuous inputs [28]. Some important examples of decision trees include extreme gradient boosted trees and random forest as they are considered as best for classification and regression purposes [29].

The models used in this study are extreme gradient boosting decision trees, random forest decision trees, and artificial neural networks (ReLu networks), each is explained briefly in the following with details about the model structure. The implementation details used for this study are also provided. All models have been developed using SKlearn, Keras, and XGBoost (extreme gradient boosting) python libraries through GoogleColab platform [30].

#### 3.4.1. Extreme Gradient Boosting Trees

Extreme gradient boosting decision trees (XGBoost) is a popular data mining algorithm, which is developed based on a combination of classification and regression trees (CART) based on a continuous training process [31], and can be used for classification purposes with a categorical output variable, or for regression purposes for estimation of a numerical target. The main idea of the XGBoost algorithm is to build a classification regression tree by using all the features in the data set. During the training process, each classification and regression tree is more fitted to the errors, which represent the difference between the observed value and the predicted value of the output variables from the ML model. The tree is then compared to the previously built tree for further adjustment and error minimization. Moreover, the XGBoost algorithm is computationally affordable, has high performance in identifying the relationship between the input and output variables, avoids overfitting, and has a minimum requirement for feature engineering compared to many other ML algorithms [32]. Furthermore, one challenge with modeling dynamic systems, such as the one in this study, is a lack of model generalization. In other words, the ML model could show a high accuracy rate on the training data, and its performance deteriorates when applied to the test data. XGBoost models have shown superiority in generalization, which means improving the model accuracy on the test data [33]. Therefore, the XGBoost algorithm, as a more appropriate decision tree for regression modeling, is used in this study. In this paper, the gradient boosting decision trees were built using 500 trees.

The model structure is constructed using the default values for each ML algorithms, as well as consideration for the type of problem, regression in the case of this study. Since XGBoost has application in both regression and classification cases, the parameters for the tress and its boosting (gaining the minimum prediction error) should be considered [34], as shown in Table 3. The details of each parameter description are given in Reference [35]. As this study is addressing a regression problem, the grid search selected values for the XGBoost with the highest predictive power are 500 trees with gblinear (for regression), with a learning rate of 0.1, maximum depth of 3 (which is the optimal number of splits in each tress nods) with sub and col samples of 1. The L1 regularization is 0 as it is only used in Ridge regression, and the L2 regularization value is 1. The loss function is linear regression which calculates the sum of squared of the model error in predicting future output values in relation to the observed values in the dataset.

#### 3.4.2. Random Forests

Random forest (RF) is another ensemble of CART algorithm, which uses the bagging method for predicting a numerical output in regression problems. During the training process, first, separate CART trees are constructed, with a predicted estimation of the output variables. In the second step, all those trees are aggregated to create an average value for the output variable. Using the bagging method for developing an RF model highly reduces the variance and the prediction bias, either underestimation or overestimation, of the output target. Furthermore, RF works perfectly with non-linear data [36]. In this paper, the random forest decision trees were built using 100 trees in each forest, with 13 as the depth of each tree. These numbers were gained using the GridSearch option for choosing the optimal number and depth for the decision trees considering the data. The base algorithm for splitting tree nodes was a decision tree regressor, with a mean square error calculated for the loss function.

#### 3.4.3. Artificial Neural Networks

**Figure 5:**
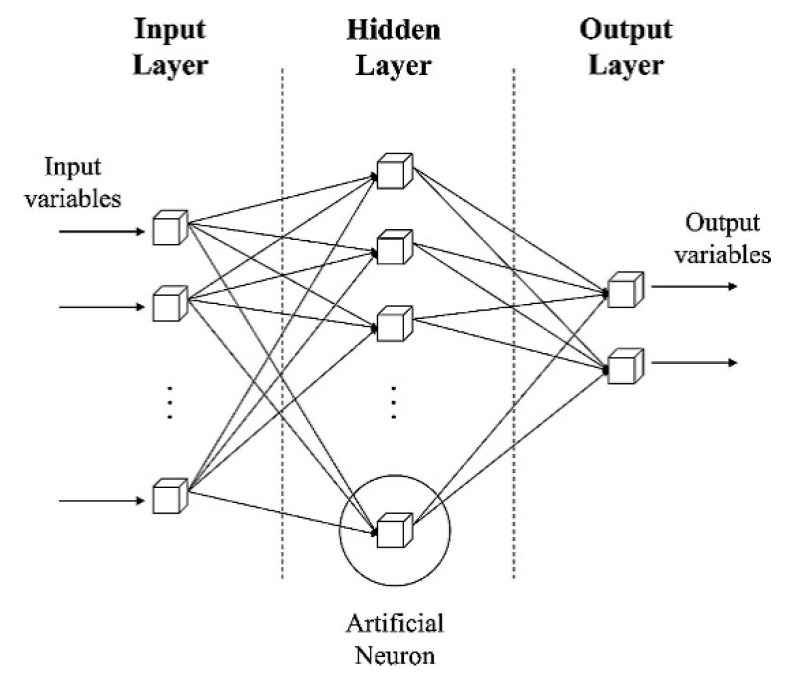
The structure of artificial neural networks (ANNs) [38].

Artificial neural networks (ANNs) is a data mining algorithm that can identify the non-linear and complex patterns in the data, and figure out the relationship between the input and output variables based on constructing a mathematical function (activation function) of inputs to minimize the error of the model in estimating an output [37]. The overall structure of ANNs consist of three layers as input, hidden, and output layers [38], as shown in Figure 5. Among various types of ANNs, such as MLP, RBF, and ReLu networks—the last one is used for this study. The reason is the compatibility of the ReLu networks with any sampling algorithms, since the training process of ReLu is not dependent on the distribution of the data [39]. The number of hidden layers, the type of activation function, the number of nodes, and the learning rate of the algorithm should be considered while developing ANNs [40].

In this paper, the structure included for the artificial neural networks one input layer, nine hidden layers, and one output layer. The model was trained for 50 epochs, with ReLu activation function, and learning rate of 0.001, using the RMSProp optimizer in Python. The details of the constructed ANN are given in Table 4.

### 3.5. Model Quantitative Performance Metrics

Model selection is a process of seeking the model in a set of candidate models that gives the best balance between model fit and complexity [41]. The comparison criterion should be based on knowledge and history of the data, as well as personal preference. 

For regression modeling, the quantitative performance metrics are mean absolute error (MAE), root mean squared error (RMSE), and R-squared (R^2^). The MAE shows the average difference between the actual values of the output variable in the original data vs. the predicted output values via the ML models. The lower the MAE, the more precise the performance of the model is in predicting future occurrences of the output. The RMSE is defined as the standard deviation of the response variable. Values of R^2^ range from 0 to 1, where 1 is a perfect fit, and 0 means there is no gain by using the model over using fixed background response rates. It estimates the proportion of the variation in the response around the mean that can be attributed to terms in the model rather than to random error. When it comes to comparing models, the one with the highest R^2^ and the lowest RMSE and MAE is preferred. The details of how MAE, RMSE, and R^2^ are calculated are shown in Table 5. 

## 4. Results

This section describes the results from XGBoost, RF, and ANNs and evaluates their performance as a surrogate for an FEA in the one-dimensional mechanical systems used in this study.

All three ML models were trained using the training data with 1.3 million data points, consisting of 80% of the total available data.

Comparing the R^2^ values, all models have high values of over 0.98. It means that the ML algorithms are able to describe over 98% of variations in the data, and are highly predictive of the *y* output based on the independent variables used in the study. Regarding the MAE and RMSE, ANNs have the lowest values of 2.11 and 8.67, respectively, followed by RF with 5.74 and 16.12 and XGBoost with 15.27 and 34.28 for MAE and RMSE values. The results suggest that ML models developed for predicting the acceleration in the beam are promising, with ANNs as the most predictive model among the three.

**Figure 6:**
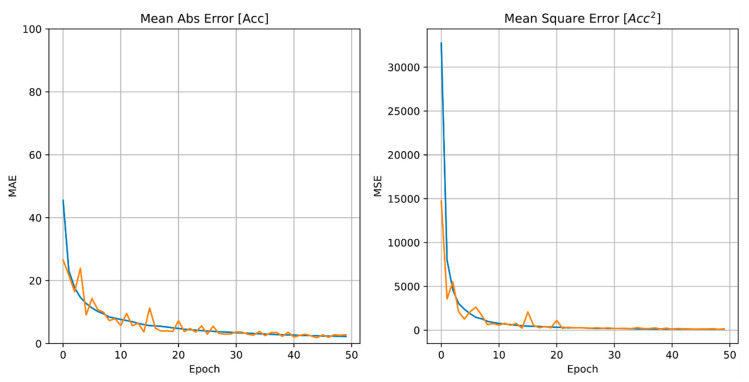
ANN MAE in predicting the acceleration variable ‘*Acc*’ased on epoch number. Orange line: Actual values; blue-line: Averaged values.

The ANN model performance, described by the mean absolute error and the mean square error, converge to an acceptable minimum after 50 epochs (Figure 6), which has therefore been chosen as providing a sufficient minimum error in predictions for this study.

**Figure 7:**
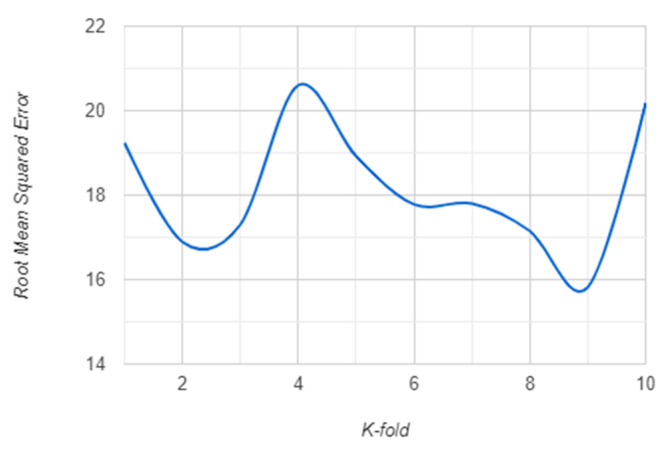
RMSE for 10-fold cross-validation of the random forest (RF) model.

**Figure 8:**
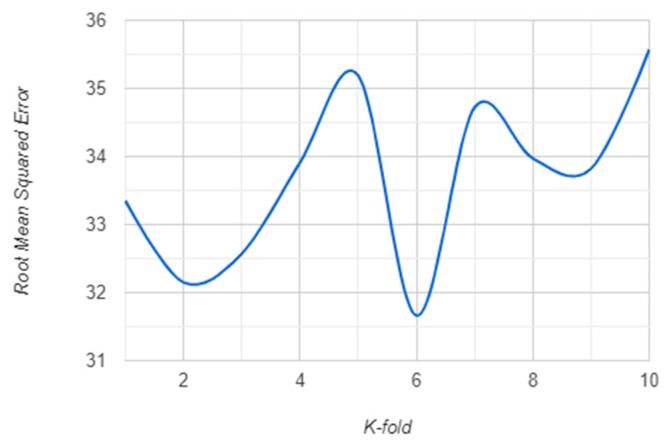
RMSE for 10-fold cross-validation of the XGBoost model.

**Figure 9:**
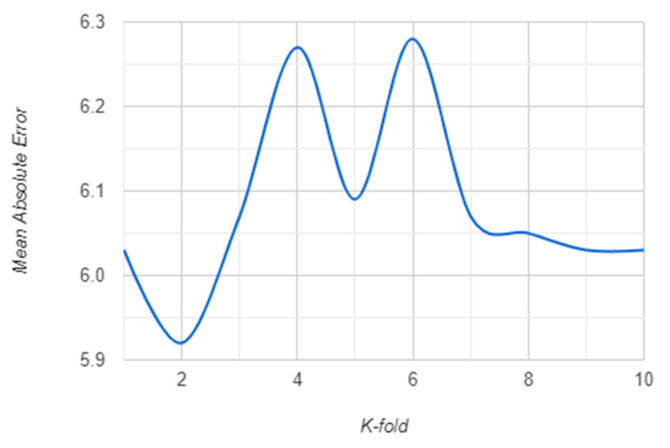
MAE for 10-fold cross-validation of the RF model.

**Figure 10:**
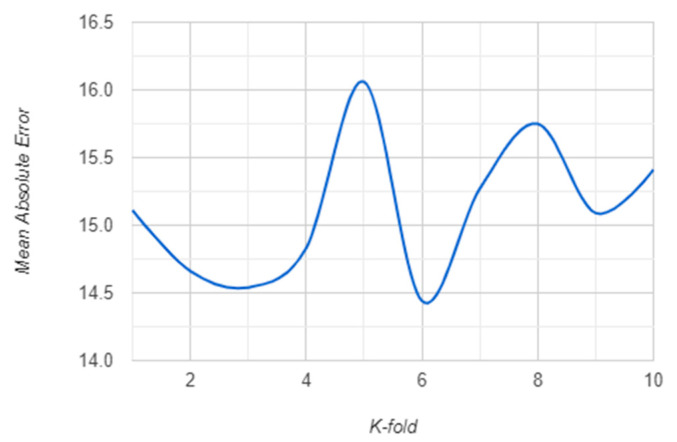
MAE for 10-fold cross-validation of the XGBoost model.

The K-fold validation error for K = {1, …, 10} for both RF and XGBoost algorithm are presented in the following: Figure 7 and Figure 8 show the RMSE behavior for each K cross-validation of RF and XGBoost. The MSE behavior is shown in Figure 9 and Figure 10 for RF and XGBoost, respectively. These plots show that the models developed using the training set can be generalized as their performance is validated by 10-fold cross-validation, both for RF and XGBoost models.

To further investigate the performance of the ML models on new data, the trained models are applied to the test data with 328,000 data points. Since these data were not used in the training part, and are new to the models, the performance of the ML models on this data is the judgement criteria for the models’ practicality when applied to a one-dimensional mechanical system. The results of the ML models’ performance on the test data are presented in Table 6. Each value in Table 6 shows the difference between the actual acceleration (output variable) in the study, and the predicted value of acceleration based on each ML model. The MAE values show that the average absolute value of the difference between the accelerations in the data, and the predicted accelerations using ANN, RF, and XGBoost are 2.87, 6.55, and 15.46 units, respectively. Therefore, ANN yielded the most precise and accurate estimation of acceleration based on the ML surrogate models. The R-squared value explains the variation in the data that can be explained and captured through the presented model. In this case, all ML algorithms are successful in explaining the variability in the data. In other words, using the input variables of this study, all three models will predict the output acceleration with over 99% fit. Looking at all three performance criteria, even though the R-squared of XGBoost is as high as RF and ANNs, it still cannot provide an accurate and precise estimation of the acceleration (MAE = 15.47 on average, and RMSE = 35.42 on average).

To illustrate the usefulness of the models in practice, the predicted values for a random set of new input variables are obtained and compared to the original values (Table 7). It shows that the predicted vs. real output values are very close, and thus, the ML models provide a close and accurate prediction of the stress in this study. With input variables in each row, the predicted values from the models are compared to the actual output values, and that is how MAE, RMSE, and R^2^ values are calculated using equations in Table 5.

As shown in Table 7, a weak pointwise accuracy is achieved by all the proposed algorithms. The ANN method is superior in achieving a strong pointwise accuracy with respect to the Random Forest and the XGBoost method. In the case of the ANN approach, a strong pointwise accuracy is achieved at all locations in Table 7, except at location x = 0.1028 m, at which the actual acceleration is small. It can be concluded that a strong pointwise accuracy is lacking only when the absolute value of the predicted variable is in the vicinity of zero, with respect to the other quantities, which is a common behavior in dynamics given the comparable signal to error values.

**Figure 11:**
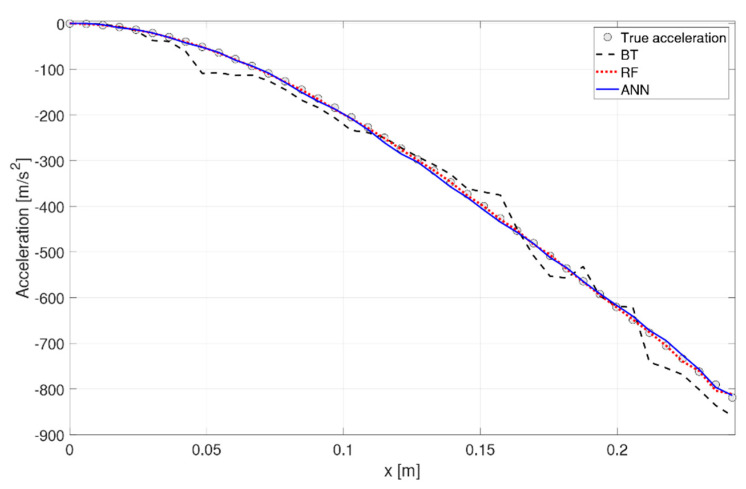
Deformed shape at t = 3.7498 s.

**Figure 12:**
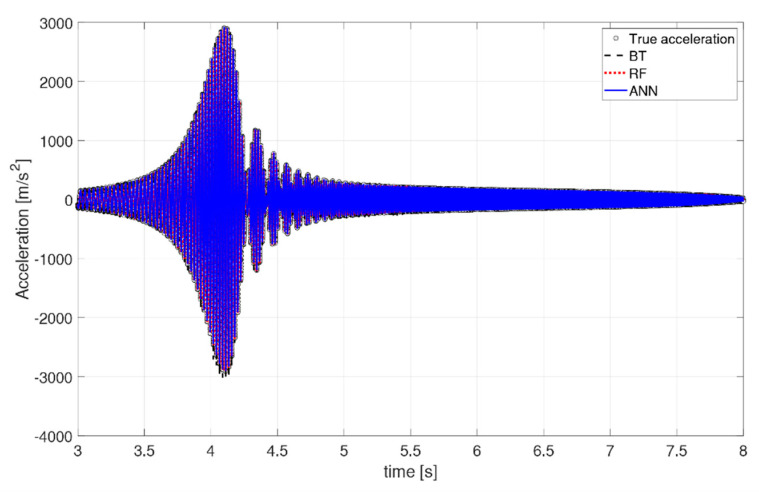
Snapshot of time history at x = 0.2057 m.

**Figure 13:**
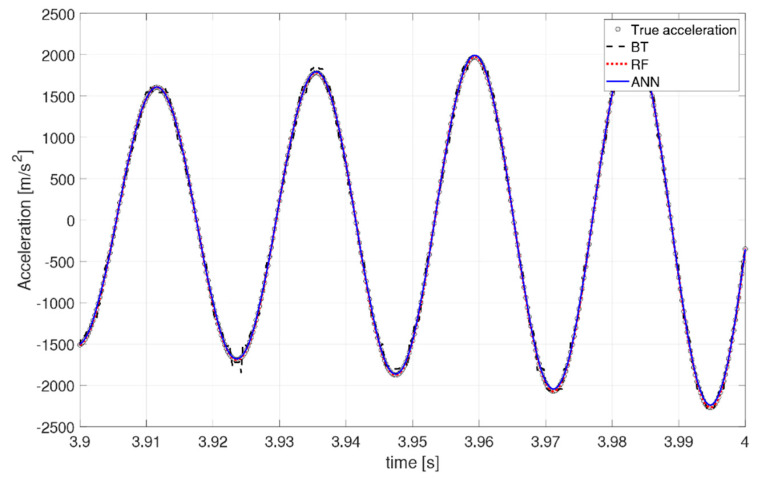
Zoom of time history at x = 0.2057 m.

It is valuable to observe how the predicted acceleration varies along the beam and with respect to time, to gauge a physical understanding of the ML performance. Specifically, Figure 11 shows the deformed shape along the beam at time instant t = 3.7498 s in, and Figure 12 and Figure 13 depict two snapshots of the acceleration varying with time at node 35 (x = 0.2057 m), respectively. The behavior of the algorithms is similar at any other location or time instant. Overall, all three algorithms behave well in their predictions. The RF and ANN algorithms are able to predict a smooth and accurate deformed shape over the entire beam, with great accuracy at every location. They also have no difficulties in predicting the time history of the system both in terms of magnitude and phase. The XGBoost algorithm seems to have more difficulties in predicting a smooth deformed shape (Figure 11), as well as the peaks in the time-varying response (Figure 12), but its overall behavior is acceptable as a mid-fidelity FEA surrogate, confirming the statistical indicators. The boundary conditions of a clamp at the root are well respected by all algorithms.

## 5. Discussion

The results, shown in Table 6 and Table 7, confirm that the ML models, when developed properly, could be used as a surrogate for an FEA considering their high accuracy and precision in predicting an acceleration in a given location based on previous acceleration at a time instant. However, this conclusion does not hold for the XGBoost model. XGBoost is a new and highly popular method that works for many regression cases successfully. One outcome of this study is that when dealing with dynamic beam data, due to its nature and the up/down variability in the values over time, XGBoost fails to estimate the acceleration values with the same high accuracy and low error of the ANNs, and RF models.

The ML algorithms, in particular RF and ANN models, can accurately predict the values of the accelerations at any time instant and spatial location. The surrogate FEA algorithms have some difficulties in predicting the initial response of the system as the numerical FEA Newmark time integrator is still converging to a numerical solution.

The main challenge in the use of ML algorithm as a surrogate finite elements lays in the necessity of large training sets, consisting of millions of data entry and must encompass the expected loading conditions that the dynamic system will encounter. In addition, additional analysis is required to establish the optimal locations for the reference locations.

The results presented in Section 4 demonstrate that the approach is feasible for one-dimensional applications. This consideration is promising for more complex applications, such as the analysis of two-dimensional and three-dimensional cases. The authors expect that the approach will be scalable to more complex systems; however, higher dimensions cases will require a larger training dataset, due to additional complexity in the model. The next steps of the research will involve the analysis of higher dimensions cases and their validation.

The results of the surrogate model confirm that it will be possible to use this method as a mid-fidelity FEA. We envision that during the design phase of a fleet of vehicles, the surrogate model will be trained to encompass the expected design loads during the lifetime of the vehicles in the fleet. During operations, each vehicle will record the response at the reference points, which will act as the input for the surrogate model. Using the trained model, it will be computationally inexpensive to obtain the response at every location in the system at every instant of time for every vehicle. The system behavior can be tracked at any time with a sufficiently accurate estimate.

## 6. Conclusions

This paper presents a novel approach to use ML algorithms as a surrogate in finite element models, in order to predict the behavior of a time-varying mechanical system. After an extensive literature review, reviewing the current state-of-the-art research on the topic, a novel algorithm combining finite element approaches and ML algorithms for transient systems is described. The approach is numerically validated for a one-dimensional system. The overall response is predicted by the ML algorithms based on the real-time response at a few reference locations. The ML models are trained based on the FEA, and then they predict the overall mechanical state of the beam at any time instant based on the response of a few points, eliminating the need to re-run an additional FEA. The performance of three ML algorithms has been evaluated in a one-dimensional framework: Gradient boosting regression trees, random forest, and artificial neural network. All three algorithms have proven as valid candidates as mid-fidelity surrogate models for an FEA, with artificial neural networks providing more accurate results. The performance of the artificial neural network is also tested to predict variables different than reference variables, and the surrogate model is shown to be flexible and accurate. The results of the surrogate model, therefore, confirm that it will be possible to use this method as a mid-fidelity FEA. We envision that during the design phase of a fleet of vehicles, the surrogate model will be trained to encompass the expected design loads during the lifetime of the vehicles in the fleet. During operations, each vehicle will record the response at the reference points, which will act as the input for the surrogate model. Using the trained model, it will be computationally inexpensive to obtain the response at every location in the system at every instant of time for every vehicle. The system behavior can be tracked at any time with a sufficiently accurate estimate.

## Figures and Tables

**Table 1:** Tabular setup of the variables for training of the machine learning algorithm.

T [s]	Nodal location [m]	Acceleration at node—Acc [m/s^2^]	*p*_1_ [m/s^2^]	*p*_2_ [m/s^2^]	*p*_3_ [m/s^2^]	*p*_4_ [m/s^2^]	*p*_5_ [m/s^2^]

**Table 2:** Descriptive statistics of the generated data used in the study.

Descriptive Statistics	Variables in the Data Set							
	Time [s]	*x* [m]	*Acc* [m/s^2^]	*p*_1_ [m/s^2^]	*p*_2_ [m/s^2^]	*p*_3_ [m/s^2^]	*p*_4_ [m/s^2^]	*p*_5_ [m/s^2^]
count	1,640,041	1,640,041	1,640,041	1,640,041	1,640,041	1,640,041	1,640,041	1,640,041
Mean	5	0.121	0.000134	0.003443	−0.00443	−0.01435	0.014168	−0.03374
Standard Deviation	2.886824	0.071585	460.3932	13.78769	433.0268	597.488	334.7891	915.9048
Minimum	0	0	−10800.2	−253.55	−5490.93	−7413.91	−2465.33	−10800.2
25% Quantile	2.5	0.0605	−34.3779	−0.24257	−30.1772	−63.572	−70.5374	−95.8869
50% Quantile	5	0.121	−1.61 × 10^−167^	−0.00023	−0.00323	−0.02278	0.114156	0.111756
75% Quantile	7.5	0.1815	34.10027	0.243119	30.05224	63.17301	70.17889	95.27278
maximum	10	0.242	10,590.98	285.8167	5576.689	7521.675	2464.305	10,590.98

**Table 3:** Details for XGBoost (extreme gradient boosting) structure by grid search.

Parameter	Description	Range in Grid Search
Booster	Boosting algorithm used in building trees	{‘gblinear, ‘gbtree’}
N_estimator	Number of boosted tress	{100, 200, 300, 400, 500}
Max_depth	Maximum tree depth for base learner	{3, 4, 5, 6, 7, 8}
Subsample	Subsample ration of the training instances	{0.5, 1}
Colsample	Subsample ratio of columns when building each tree	{0.5, 1}
Reg_alpha	L1 regularization term on weights (used for Ridge regression)	{0.01, 0.5, 1}
Reg_lambda	L2 regularization term on weights (used to reduce high dimension)	{0.01, 0.5, 1}
Learning_rate	Boosting learning rate	{0.01, 0.05, 0.1, 0.2}

**Table 4:** Details for ANN structure in this study.

Layer	Structure Criteria	ReLu Structure
Input Layer	Factors	position x; p_i_(t)
	Number of Units	6
Hidden Layer	Number of Hidden Layers	9
	Number of Units	1952
	Activation Function	Rectified Linear Unit (ReLU)
Output Layer	Dependent Variables	acceleration
	Number of Units	1
	Error Function	Mean Squared Error

**Table 5:** Model comparison criteria.

Criterion	Formula
MAE *	$\frac{1}{n}\overset{n}{\underset{i=1}{\sum }}\left|yi-\hat{y}i\right|$
RMSE *	$\sqrt{\overset{n}{\underset{i=1}{\sum }}\frac{{\left(yi-\hat{y}i\right)}^{2}}{n}}$
R2	$1-\frac{\sum_{i=1}^{n}{\left(yi-\hat{y}i\right)}^{2}}{\sum_{i=1}^{n}{\left(yi-\bar{y}i\right)}^{2}}$

* MAE, mean absolute error; RMSE, root mean square error; *n*, number of data points used in the model; *n*, number of observations in the data set; yi, the real value for the output variable; $\hat{y}i$, predicted value for the output variable; $\bar{y}i$, average value for all output variables in the data set.

**Table 6:** Model performance on test data.

Model Performance Metric	ANNs	RF	XGBoost
Mean Absolute Error	2.87	6.55	15.47
Root Mean Squared Error	10.58	19.62	35.42
R-squared	0.9994	0.9981	0.9940

**Table 7:** Predicted values for acceleration “Acc” for a set of input variables from the test data. The data in the table was randomly selected to show models’ performance.

Input Variables						Output	Predicted Output Per Model		
X [m]	p1 [m/s^2^]	p2 [m/s^2^]	p3 [m/s^2^]	p4 [m/s^2^]	p5 [m/s^2^]	Actual Acc [m/s^2^]	Acc ANNs [m/s^2^]	Acc RF [m/s^2^]	Acc XGBoost [m/s^2^]
0.0847	−0.273	−39.709	−84.76	−132.28	−160.617	−51.63	−51.59 0.08%	−52.36 1.42%	63.00 16.9%
0.0847	−0.50	−70.812	−129.20	−110.034	−96.189	−89.79	−90.67 0.99%	−91.84 2.29%	69.18 32.7%
0.0121	−3.162	−397.07	−985.0	−2192.01	−2883.29	−12.48	−13.95 11.74%	−12.858 3.02%	11.047 16.40%
0.1331	0.086	10.4072	26.542	62.195	82.48	31.49	33.09 5.06%	35.19 11.7%	45.39 22.5%
0.0363	−5.106	15.067	11.276	−56.605	−111.163	6.109	7.146 16.9%	11.34 85.6%	4.243 167%
0.1391	0.164	21.744	51.713	105.605	136.828	63.76	64.86 1.74%	65.09 2.09%	68.59 5.11%
0.1996	0.030	3.594	9.2598	22.098	29.38	22.10	22.38 1.29%	22.348 1.13%	29.01 22.9%
0.1512	0.028	3.306	8.5029	20.230	26.889	12.73	14.0 9.96%	14.94 17.3%	11.658 28.1%
0.1028	0.0029	0.3776	0.9086	1.90	2.4729	0.697	1.715 145%	1.98 184%	3.12 350%
0.0544	0.081	9.763	25.016	59.115	78.494	5.721	5.449 4.75%	6.64 16.1%	4.97 33.8%
0.0242	−1.283	−141.21	−221.38	−50.023	82.482	−19.73	−19.19 2.74%	−19.33 2.04%	20.17 4.14%

## Data Availability

Data available on request.

## References

[1] Smarsly K., Lehner K., Hartmann D. (2007). Structural Health Monitoring based on Artificial Intelligence Techniques. Comput. Civil Eng..

[2] Chierichetti M., Grappasonni C., Coppotelli G., McColl C. (2014). A modal approach for dynamic response monitoring from experimental data. Mech. Syst. Signal Process..

[3] Chierichetti M., McColl C., Ruzzene M. (2014). Prediction of UH-60A blade loads: Insight on load confluence algorithm. AIAA J..

[4] Chierichetti M. (2014). Load and response identification for a nonlinear flexible structure subject to harmonic loads. J. Comput. Nonlinear Dyn..

[5] Liang L., Liu M., Martin C., Sun W. (2018). A deep learning approach to estimate stress distribution: A fast and accurate surrogate of finite-element analysis. J. R. Soc. Interface.

[6] Curnow W.J. (2003). The efficacy of bicycle helmets against brain injury. Accid. Anal. Prev..

[7] McColl C., Palmer D., Chierichetti M., Bauchau O., Ruzzene M. Comprehensive UH-60 loads model validation. Proceedings of the 66th American Helicopter Society International Annual Forum-AHS International.

[8] Alpaydin E. (2020). Introduction to Machine Learning.

[9] Madani A., Bakhaty A., Kim J., Mubarak Y., Mofrad M.R.K. (2019). Bridging Finite Element and Machine Learning Modeling: Stress Prediction of Arterial Walls in Atherosclerosis. J. Biomech. Eng..

[10] Martin-Guerrero J.D., Ruperez-Moreno M.J., Martinez-Martinez F., Lorente-Garrido D., Serrano-Lopez A.J., Monserrat C., Martinez-Sanchis S., Martinez-Sober M. Machine Learning for Modeling the Biomechanical Behavior of Human Soft Tissue. Proceedings of the IEEE International Conference on Data Mining Workshops, ICDMW.

[11] Martínez-Martínez F., Rupérez-Moreno M.J., Martínez-Sober M., Solves-Llorens J.A., Lorente D., Serrano-López A.J., Martínez-Sanchis S., Monserrat C., Martín-Guerrero J.D. (2017). A finite element-based machine learning approach for modeling the mechanical behavior of the breast tissues under compression in real-time. Comput. Biol. Med..

[12] Lorente D., Martínez-Martínez F., Rupérez M.J., Lago M.A., Martínez-Sober M., Escandell-Montero P., Martínez-Martínez J.M., Martínez-Sanchis S., Serrano-López A.J., Monserrat C. (2017). A framework for modelling the biomechanical behaviour of the human liver during breathing in real time using machine learning. Expert Syst. Appl..

[13] Koutsourelakis P.S. (2007). Stochastic upscaling in solid mechanics: An excercise in machine learning. J. Comput. Phys..

[14] Capuano G., Rimoli J.J. (2019). Smart finite elements: A novel machine learning application. Comput. Methods Appl. Mech. Eng..

[15] Oishi A., Yagawa G. (2017). Computational mechanics enhanced by deep learning. Comput. Methods Appl. Mech. Eng..

[16] Kirchdoerfer T., Ortiz M. (2016). Data-driven computational mechanics. Comput. Methods Appl. Mech. Eng..

[17] Raissi M., Perdikaris P., Karniadakis G.E. (2019). Physics-informed neural networks: A deep learning framework for solving forward and inverse problems involving nonlinear partial differential equations. J. Comput. Phys..

[18] Raissi M., Perdikaris P., Karniadakis G.E. (2017). Physics Informed Deep Learning (Part II): Data-driven Discovery of Nonlinear Partial Differential Equations. arXiv.

[19] Raissi M., Karniadakis G.E. (2018). Hidden physics models: Machine learning of nonlinear partial differential equations. J. Comput. Phys..

[20] Mao Z., Jagtap A.D., Karniadakis G.E. (2020). Physics-informed neural networks for high-speed flows. Comput. Methods Appl. Mech. Eng..

[21] Martinez D., Sitaraman J., Brewer W., Rivera P., Jude D. Machine Learning Based Aerodynamic Models For Rotor Blades. Proceedings of the Aeromechanics for Advanced Vertical Flight Technical Meeting.

[22] Gao W., Lu X., Peng Y., Wu L. (2020). A Deep Learning Approach Replacing the Finite Difference Method for In Situ Stress Prediction. IEEE Access.

[23] Rahneshin V., Chierichetti M. (2016). An integrated approach for non-periodic dynamic response prediction of complex structures: Numerical and experimental analysis. J. Sound Vib..

[24] Chierichetti M., Davoudi F., Huang D., Vurturbadarinath P., Linzmeyer M. (2021). Surrogated Finite Element Models Using Machine Learning.

[25] Chierichetti M., Ruzzene M. (2011). Model updating in structural dynamics through a confluence algorithm. J. Theor. Appl. Mech..

[26] Chierichetti M., Demetriou M.A., Zonta D., Huang H. (2020). Moving sensors in structural dynamics. Proceedings of the Sensors and Smart Structures Technologies for Civil, Mechanical, and Aerospace Systems 2020.

[27] Cook R.D., Malkus D.S., Plesha M.E., Witt R.J. (2001). Concepts and Applications of Finite Element Analysis.

[28] Abbott D. (2014). Applied Predictive Analytics. Principles and Techniques for the Professional Data Analyst.

[29] Cui Z., Chen W., He Y., Chen Y. Optimal Action Extraction for Random Forests and Boosted Trees. Proceedings of the 21th ACM SIGKDD international conference on knowledge discovery and data mining.

[30] Welcome To Colaboratory-Colaboratory. https://colab.research.google.com/github/prites18/NoteNote/blob/master/Welcome_To_Colaboratory.ipynb.

[31] Wu Z., Wang X., Jiang B. (2020). Fault diagnosis for wind turbines based on ReliefF and eXtreme gradient boosting. Appl. Sci..

[32] Dong W., Huang Y., Lehane B., Ma G. (2020). XGBoost algorithm-based prediction of concrete electrical resistivity for structural health monitoring. Autom. Constr..

[33] Wang S., Liu S., Zhang J., Che X., Yuan Y., Wang Z., Kong D. (2020). A new method of diesel fuel brands identification: SMOTE oversampling combined with XGBoost ensemble learning. Fuel.

[34] Zhou Y., Li T., Shi J., Qian Z. (2019). A CEEMDAN and XGBOOST-Based Approach to Forecast Crude Oil Prices. Complexity.

[35] XGBoost Parameter—Xgboost 1.4.0-SNAPSHOT Documentation. https://xgboost.readthedocs.io/en/latest/parameter.html.

[36] Liu J., Sun S., Tan Z., Liu Y. (2020). Nondestructive detection of sunset yellow in cream based on near-infrared spectroscopy and interval random forest. Spectrochim. Acta Part A Mol. Biomol. Spectrosc..

[37] Ahmadi M.A., Ebadi M., Hosseini S.M. (2014). Prediction breakthrough time of water coning in the fractured reservoirs by implementing low parameter support vector machine approach. Fuel.

[38] Jeon J.H., Yang S.S., Kang Y.J. (2020). Estimation of sound absorption coefficient of layered fibrous material using artificial neural networks. Appl. Acoust..

[39] Grimstad B., Andersson H. (2019). ReLU networks as surrogate models in mixed-integer linear programs. Comput. Chem. Eng..

[40] Kakhki F.D., Freeman S.A., Mosher G.A. (2019). Use of neural networks to identify safety prevention priorities in agro-manufacturing operations within commercial grain elevators. Appl. Sci..

[41] Burnham K.P., Anderson D.R. (2004). Multimodel inference: Understanding AIC and BIC in model selection. Sociol. Methods Res..

